# The Book World of Medicine and Science

**Published:** 1905-07-29

**Authors:** 


### The Book World of Medicine and Science.

Handbook of Intestinal Surgery. By Leonard A. Bidwell, F.R.C.S. (London: Baillière, Tindall and Cox. 1905. Pp. 167. Price 6s. net.)

Commencing with a careful and well-illustrated description of the varieties of intestinal suture available, Mr. Bidwell proceeds to discuss their application in special operations. These considerations occupy the first seven chapters, the remainder of the volume consisting of three chapters on abdominal incisions, the preparation, and the after-treatment of abdominal cases. The chief portion of the book is admirable, and affords but little opportunity for any comment except praise. It is a pity, however, that the last two chapters were included in the work. They are characterised equally by superfluities and omissions, and this is all we have to say of them. The author's advice on p. 90 to reduce gently a piece of strangulated intestine whose vitality is doubtful, and leave it near the hernial opening, is open to the objection that reduced bowel will not always remain in the desired place unless it is fastened there. With regard to the use of morphia, Mr. Bidwell does not think that an injection preliminary to an operation is a safe procedure, and adds that it increases the ultimate shock. This opinion is difficult to accept, and we should be very glad to know upon what facts it is based. It should be stated, in view of the comprehensive title, that the author limits himself almost entirely to the technique of intestinal surgery, and does not discuss symptoms, indications for operation, after results, and so forth. To conclude our criticism we will say that, taken as a whole, the work is of a high standard and full of instruction, and it ought to be in the possession of every operating surgeon.

Homœopathy Explained. By John Henry Clarke, M.D. (London Homœopathic Publishing Company. 1905. Pp. 212.)

The medical profession, as a rule, may be said to regard homœopathy as a system the followers of which differ from legitimate practitioners of medicine chiefly by the use of an esoteric jargon, fully intelligible only to each other, and chiefly useful as a support to the belief, still existing among a certain section of the public, that there is something peculiar in the principles by which the so-called Homœopathists are guided, and in the details of the practice which they pursue. The fact that homœopathists are, as a rule, legally qualified medical practitioners shows that they must have received an ordinary medical education, and that they have at least no excuse for being ignorant of the principles upon which that education was based; while the additional fact that, also as a rule, they are not known to be, on the average, less successful than their non-homœopathic competitors, points distinctly to their doing the same things very much in the same way, and to their general attainment of much the same practical results. In a word, they are believed to employ precisely the same methods of diagnosis, and the same principles of treatment, as practitioners who refuse to be ticketed with an appellation. To the present generation Hahnemann, like Junius, is no more than *nominis umbra*. Dr. Clarke is dissatisfied with this estimation of his fraternity, and so he has written a little book containing 192 pages of letterpress and 20 pages of index, and intended to prove that Hahnemann was a man of science and a discoverer, and that his system of so-called homœopathy has a real basis in fact and in experience. As a scientific production the book is a tissue of absurdities, but it is not badly contrived as a means of impressing that portion of the ignorant public which derives conceptions of disease and of remedies from the columns of the inferior non-medical Press.

Tropical Light. By Major Chas. E. Woodruff, A.M., M.D. (London: Rebman Company, 1905. Pp. 358. Price 10s. 6d. net.)

Major Woodruff has produced a book which is of extreme interest. He says it was written to prove, or disprove, the hypothesis of Von Schmaedel that the pigmentation of the skin in man was evolved to protect him from the actinic rays of light. He starts by showing how the earth is divided into zones with equalised temperatures, and how both men and animals are adjusted to their environment, and migration sooner or later means extinction of the species. But he holds that the evolution not only has to do with heat, but also, and to a greater degree, with light. It was by studying the effects of actino-therapy that he first became convinced that Von Schmaedel's theory was correct; that it is the violet and ultra-violet rays that are harmful; and that pigmentation was evolved to protect man from these. The first action of this light is that of a powerful stimulant. This stimulation if carried to excess is followed by a reaction which is equally marked. This is a source of great danger to people on first going to the tropics; led away by a false idea, they do more work than is right, and unnecessarily expose themselves. His study convinces him that the blonde, other things being equal, is less fitted for the tropics than the brunette. The last chapter is devoted to rules for white men in the tropics, some of which are new, but probably none the less to be observed.

Health and Disease in Relation to Marriage and the Married State. Vol. II. Edited by Dr. Senator and Dr. Kaminer. Translated into English by J. Dulberg, M.D. (London: Rebman, Limited, 1905.)

In our review of the former volume of this excellent work, we had occasion to point out how much such a book was needed, and how well the editors had carried out their task, and it is not necessary to say more about the present volume than that it is quite equal to its predecessor. It is a storehouse of information, and is certain to be a standard work of reference on the problems of heredity, pathology, and psychology, in their relation to marriage and the married state.

Golden Rules of Medical Practice. By Lewis Smith, M.D., M.R.C.P. No. IV. (Bristol: J. Wright. 1905. Pp. 126.)

We have on several occasions reviewed different numbers of the "Golden Rules" series. The fourth in the series—Golden Rules of Medical Practice—has how reached the sixth edition. Having been enlarged and entirely rewritten, it has increased in merit and value, and will be found especially useful to those commencing general practice.

Adenoid Growths of the Naso-Pharynx. By G. A. Garry Simpson, M.R.C.S. (London: Messrs. Bale, Sons, and Danielsson, 1904. Pp. 43. 16 Illustrations. Price 3s. 6d.)

This is a clearly written and well illustrated little book. A very fair and moderate view is taken of the subject, but there is nothing new or original about the work.

